# Diabetes research enters the biobank era: searching for the truth in a deep well

**DOI:** 10.1172/JCI199728

**Published:** 2025-12-01

**Authors:** Decio L. Eizirik, Priscila L. Zimath

**Affiliations:** ULB Center for Diabetes Research, Medical Faculty, Université Libre de Bruxelles (ULB), Brussels, Belgium.

## Abstract

Loss of circulating insulin resulting from autoimmune destruction of β cells is the defining characteristic of type 1 diabetes (T1D), but islet dysfunction in T1D affects both β cells and α cells. Advances in multiomic analyses and the systematic collection of diseased human pancreata are enabling new approaches for diabetes research; hypotheses can be generated from observations in the affected human tissue and then tested in human islets, stem cell–derived islets, or humanized mice. The study by dos Santos and colleagues that appears in this issue of the JCI is an excellent example of the advantages and challenges posed by this approach. Through integrated analyses that combined electrophysiological and transcriptomic profiling, the authors provided detailed insights into the mechanisms leading to α cell dysfunction in islets from individuals with T1D.

## Advantages of genome-based studies of donor pancreatic islets

In past decades, research on β cell fate in diabetes usually started with hypothesis generation and testing in rodent models and then, if feasible, proceeded to confirmation of key findings in human islets. Trust by some scientists in these rodent-based conclusions and in their own convictions were so “rock solid” that, often, a single experiment in human islets was considered sufficient to “prove” the rodent model findings.

A classic example of this era was the so called “Okamoto Model”; based on experiments in rodent islets exposed to the β cell toxins streptozotocin and alloxan, this model posited that activation of poly(ADP–ribose) synthetase (PARP) and consequent NAD depletion (preventable by nicotinamide) was the central cause of β cell death in type 1 diabetes (T1D) (1, 2). The Okamoto Model laid the foundation for a large double-blind, placebo-controlled trial testing nicotinamide as an approach to prevent T1D in 552 individuals with islet cell antibodies (ICAs) (the ENDIT trial (3)). Unfortunately, the results of this and subsequent trials based on a similar framework were negative (4). By then, it had started to dawn on many scientists that the field faced a phenomenon akin to that described by Thomas Mann in the introduction to his masterpiece “Joseph and His Brothers” (5), discussing the study of ancient history: “And so one runs the risk, when peering down into the well of the past, of seeing at the bottom not the real past … but rather one’s own face” — or, in the present case, one’s own favorite rodent-based hypothesis.

**Figure 1:**
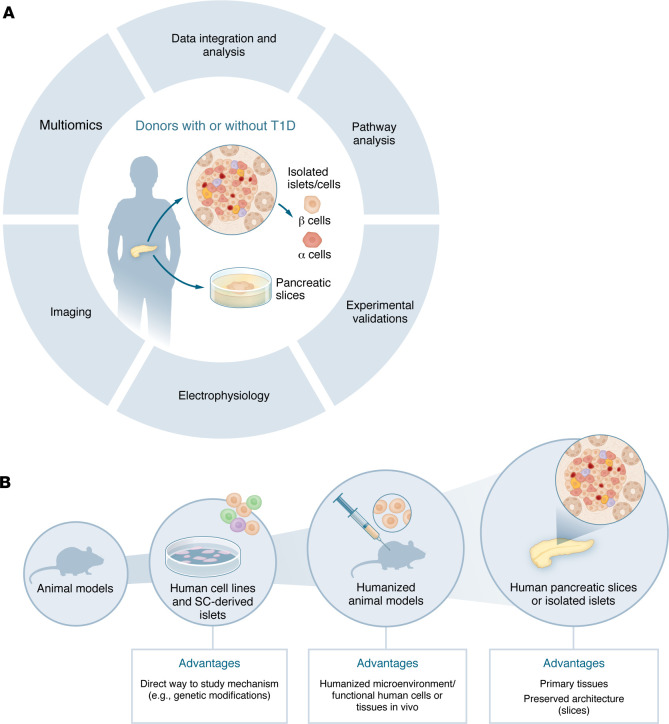
A paradigm shift in diabetes research: from “rodent-first” to “human-based” approaches. Traditional diabetes research often followed a “rodent-first” paradigm, where hypotheses were generated and tested in animal models and only then validated in human islets. Advances in genomics, proteomics, and systematic human pancreatic tissue collection have enabled a “human-first” approach, where hypotheses are now generated from affected human tissue at its full complexity (**A**) and subsequently tested in human β-cell lines, islets, stem cell–derived organoids, or humanized mouse models (**B**).

The solution to this conundrum arrived from 2 sides. First, the human genome project enabled the development of tools to comprehensively profile mRNAs and proteins, initially with microarrays, later with RNA-seq, advanced proteomics, and, more recently, spatial transcriptomics and proteomics. Use of genome-based tools shifted thinking from the traditional “one gene, one protein, one disease” view to understanding that complex conditions, such as β cell failure in diabetes, often result from perturbed gene and protein networks. Second, American and European initiatives, such as the Network for Pancreatic Organ Donors with Diabetes (nPOD, https://npod.org/) (6), Human Pancreas Analysis Program (HPAP, https://hpap.pmacs.upenn.edu/) (7) and Exeter Archival Diabetes Biobank (EADB, https://foulis.vub.ac.be) (8), systematically collected pancreata from nondiabetic individuals and individuals positive for islet autoantibodies or with clinical T1D. These tissues were analyzed by advanced omics and/or histology, and data were deposited in open access resources (similar initiatives were developed for brain and other tissues [e.g., Human BioMolecular Atlas Program (HuBMAP) (9), Human Protein Atlas (HPA) (10), etc.]). Together, these advances enabled a different approach to human diabetes research; hypotheses could now be generated departing from the affected human tissue — which could be studied at its full complexity (Figure 1A) — and then tested in human β cell lines, islets, or stem cell-derived islet organoids, humanized mice, or spontaneous animal models of diabetes, such as NOD mice (Figure 1B).

## Comparing α cell and β cell dysfunction in T1D islets

The study by dos Santos and colleagues (11) published in this issue of the JCI is an excellent example of the advantages and challenges posed by this new approach. Through integration of electrophysiological analyses and transcriptomic profiling in donor islet cells, the authors provide new insights into β and α cell dysfunction in individuals with and without T1D (Figure 1A). In T1D islets, surviving β cells were scarce (16 cells from 4 donors), reflecting longstanding disease. Indeed, only one T1D islet was studied at the time of the donor’s diagnosis — all of the other donors had had diabetes for 3–41 years. This explains the β cell scarcity and should caution against bias when interpreting these results: the study of a few surviving β cells that endured years of autoimmune attack limits the relevance for understanding early β cell events in the development of T1D. The surviving β cells in T1D islets showed impaired electrophysiological activity, transcriptional signatures of increased antigen presentation (increased MHC class I– and IFN-γ–related pathways, both key in T1D pathogenesis), a shift towards glycolysis, downregulation of mitochondrial respiration, and impaired protein translation relative to β cells from nondiabetic islets changes suggesting metabolic reprogramming of these immune-stressed cells. These findings align with previous studies in pancreatic slices showing that residual β cells in T1D are functionally impaired (12, 13).

In contrast, α cells (569 cells from 9 donors with T1D) were preserved but exhibited hyperexcitability, increased exocytosis, and loss of physiological glucose-mediated suppression of glucagon secretion, paralleled by dysregulation of mTORC1 complex signaling, compared with α cells from nondiabetic islets. Studying α cells in T1D is important, as these cells contribute to dysregulation of glucose homeostasis, initially by aggravating hyperglycemia due to glucagon hypersecretion and, at later stages of the disease, by failing to provide counter regulation to hypoglycemia (14, 15). The functional alterations in T1D islet α cells were accompanied by upregulation of immune signaling transcripts (e.g., HLA-A/B/C and B2M), dysregulation of mTOR signaling, and lysosomal imbalance, all of which likely contribute to aberrant glucagon release. Microscopy analysis revealed compartmentalization of glucagon and MHC-I in α cells from nondiabetic individuals, but colocalization in T1D α cells. Based on these findings, dos Santos et al. suggested that the negative correlation between the α cell score (an integrated metric for α cell behavior) and antigen presentation genes (HLA-A and -B, B2M, etc.) in T1D islets indicates that upregulation of antigen loading and presentation somehow affects α cell function. HLA expression in islet cells is one of the best markers of interferon exposure (16, 17). Thus, an alternative explanation could be that cells that faced greater exposure to interferons — resulting in higher HLA class I expression — had a lower α cell score. In other words, higher HLA class I expression and lower α cell score could be concurrent to interferon exposure, without implying causality.

## Connecting T1D genetic risk variants to α cell dysfunction

Another interesting and counterintuitive observation in dos Santos et al.’s report was the upregulation of key transcription factors required for maintenance of α cell phenotype, such as NeuroD1 and ISL1, in individuals with diabetes. This, however, was paralleled by a general impairment in nuclear access, which may result in defective action of the transcription factors. In future studies in α and β cells, it would be of interest to perform single-cell RNA-seq and single-nuclear RNA-seq in parallel to determine whether this defective nuclear transfer is specific for some transcription factors or is a general phenomenon.

By linking electrophysiological phenotypes to molecular signatures, this study showed that α cell dysfunction genes and pathways were enriched for T1D genetic risk variants (derived from the T1D Knowledge Portal and islet expression quantitative trait loci [eQTL]) (18). Although T1D genetic risk is classically linked to T cell activity and β cells (19), dos Santos and colleagues’ findings now extend this to α cells. The data suggest that α cell dysfunction is shaped by a combination of genetic, transcriptomic, and biophysical factors: cells with pronounced electrophysiological impairment expressed higher levels of T1D risk genes, while cells with preserved function showed reduced expression. Three points to consider here are that: (a) most risk variants for autoimmune diseases act in noncoding regions; (b) islet eQTL in the context of T1D, and differently from T2D, is better detected when islets are exposed to proinflammatory cytokines (20, 21); and (c) there are new tools available to determine islet cell eQTLs in both T1D and T2D (18). It will therefore be of interest to reanalyze the present data in light of this new information.

## Scouting mechanisms that protect α cells during β cell destruction

A feature of T1D is that the immune system destroys β cells but not neighboring α cells, even though both are dysfunctional. Although α cells are exposed to the same inflammatory environment as β cells and the cells share gene expression signatures (22, 23), α cells survive. Understanding how α cells resist the prolonged immune assault that characterizes T1D pathogenesis may uncover protective mechanisms and novel therapeutic targets to preserve β cells and prevent or delay disease. By highlighting the role of α cells, the authors provide a new and valuable perspective on T1D pathophysiology. Several other relevant T1D pathways were identified in this study, and it will provide an excellent blueprint for future studies to map α cell fate in T1D.

## Future directions and challenges of islet-based T1D research

Research in human T1D pancreata faces challenges that are not unique to the present study. The restricted availability of T1D donor tissue limits the number of islets and cells available to be analyzed; it is furthermore impossible to perform longitudinal studies, distinguishing this research from research based on sequential biopsies of skin in psoriasis or joints in rheumatoid arthritis. Another complication is donor variability in terms of disease duration, age at onset, biological sex, and autoantibody profiles; each variable introduces heterogeneity but also reflects the clinical diversity of T1D (24). To partially mitigate these challenges, alternative approaches have been used, such as studying intact pancreatic slices (13). This model preserves the islet microenvironment, minimizes the impact of enzymatic isolation, and enables us to study endocrine and immune cell crosstalk. Such cross-sectional experiments inform on a single time point only and do not capture dynamic β and α cell changes over the course of disease — a pitfall that can only be addressed in vitro using human islets and stem cell-derived islets, alone or in coculture with immune cells, or, as the methodology improves, in vivo in humanized mice (25). Integrated with complementary technologies, including patch-clamp electrophysiology, multiomics, and in vivo intravital imaging, approaches enabling study at multiple time points hold promise to address important questions in T1D pathogenesis, such as the distinct contributions of α and β cells, the persistence and functionality of surviving β cells, and the heterogeneity of disease progression.

Notwithstanding the limitations discussed above, dos Santos et al. (11) extend our knowledge of the pathogenesis of T1D, providing a detailed view of α cell dysfunction in T1D and highlighting potential pathways that contribute to T1D pathophysiology.

## Funding support

Breakthrough T1D (3-SRA-2023-1379-S-B, 3-IND-2024-1549-I-X, 2-SRA-2024-1524-S-B and IDDP-InSphero 201310390).
The Leona M. & Harry B. Helmsley Charitable Trust (Grants 2402-08172 and 2402-08172).
The Win4Excellence – Gene therapy in Wallonie (GT4Health_20230920).

## Version 1 12/01/2025

Electronic publication
